# Developing weight navigation program to support personalized and effective obesity management in primary care settings: protocol for a quality improvement program with an embedded single-arm pilot study

**DOI:** 10.1017/S1463423621000906

**Published:** 2022-03-02

**Authors:** Dina H. Griauzde, Amal Othman, Chris Dallas, Lauren Oshman, Jonathan Gabison, Dorene S. Markel, Caroline R. Richardson, Jeffrey T. Kullgren, Gretchen Piatt, Michele Heisler, Amy M. Kilbourne, Andrew Kraftson

**Affiliations:** 1 VA Ann Arbor Healthcare System, Ann Arbor, MI, USA; 2 Department of Internal Medicine, University of Michigan, Medical School, Ann Arbor, MI, USA; 3 University of Michigan, Institute for Healthcare Policy and Innovation, Ann Arbor, MI, USA; 4 Department of Family Medicine, University of Michigan, Medical School, Ann Arbor, MI, USA; 5 Department of Learning Health Sciences, University of Michigan, Medical School, Ann Arbor, MI, USA; 6 Department of Health Management and Policy, University of Michigan, School of Public Health, Ann Arbor, MI, USA; 7 Department of Health Behavior and Health Education, University of Michigan, School of Public Health, Ann Arbor, MI, USA

**Keywords:** obesity, personalized care, population health, primary care, weight loss

## Abstract

**Background:**

Primary care providers (PCPs) are expected to help patients with obesity to lose weight through behavior change counseling and patient-centered use of available weight management resources. Yet, many PCPs face knowledge gaps and clinical time constraints that hinder their ability to successfully support patients’ weight loss. Fortunately, a small and growing number of physicians are now certified in obesity medicine through the American Board of Obesity Medicine (ABOM) and can provide personalized and effective obesity treatment to individual patients. Little is known, however, about how to extend the expertise of ABOM-certified physicians to support PCPs and their many patients with obesity.

**Aim:**

To develop and pilot test an innovative care model – the Weight Navigation Program (WNP) – to integrate ABOM-certified physicians into primary care settings and to enhance the delivery of personalized, effective obesity care.

**Methods:**

Quality improvement program with an embedded, 12-month, single-arm pilot study. Patients with obesity and ≥1 weight-related co-morbidity may be referred to the WNP by PCPs. All patients seen within the WNP during the first 12 months of clinical operations will be compared to a matched cohort of patients from another primary care site. We will recruit a subset of WNP patients (*n* = 30) to participate in a remote weight monitoring pilot program, which will include surveys at 0, 6, and 12 months, qualitative interviews at 0 and 6 months, and use of an electronic health record (EHR)-based text messaging program for remote weight monitoring.

**Discussion:**

Obesity is a complex chronic condition that requires evidence-based, personalized, and longitudinal care. To deliver such care in general practice, the WNP leverages the expertise of ABOM-certified physicians, health system and community weight management resources, and EHR-based population health management tools. The WNP is an innovative model with the potential to be implemented, scaled, and sustained in diverse primary care settings.

## Background

Excess body weight (ie, overweight and obesity) is a leading cause of morbidity and mortality (Prospective Studies Collaboration, 2009) as well as a major risk factor for severe illness and death from the novel coronavirus 2019 (Popkin *et al.*, 2020). Modest weight loss of ≥5% body weight can help individuals with overweight or obesity to prevent, manage, or reverse weight-related chronic conditions (Anderson & Konz, 2001; Diabetes Prevention Program Research Group, 2009; Garvey *et al*., 2012; Rothberg *et al.*, 2017), and reduce individuals’ annual medical care costs by over $2,000 (Cawley *et al*., 2015). Due to the human and economic costs of obesity, health policies support the use of evidence-based obesity treatments (eg, weight loss surgery and lifestyle change programs) and intensive behavioral counseling by primary care providers (PCPs) (Kahan & Zvenyach, 2016). Moreover, clinical practice guidelines encourage PCPs and their practices to play central roles in obesity management (Final Recommendation Statement: Weight Loss to Prevent Obesity-Related Morbidity and Mortality in Adults: Behavioral Interventions - US Preventive Services Task Force, 2003; Jensen *et al.*, 2014), and the patient-centered medical home model provides an ideal context to deliver team-based, coordinated, and longitudinal obesity care (Bernstein *et al.*, 2016).

Despite these initiatives and opportunities, patients with obesity often receive suboptimal weight management in general practice settings due, in part, to provider- and practice-level factors (Tsai & Wadden, 2009). Such factors include most PCPs’ lack of specialized training in obesity treatment, brief office visits with competing clinical demands, and strained clinic capacities, which preclude frequent weight-focused visits (Cabana *et al*., 1999; Foster *et al.*, 2003; Tsai & Wadden, 2009; Salinas *et al*., 2011; Wadden *et al.*, 2014; Ossolinski *et al.*, 2015; Phelan *et al*., 2015; Mainous *et al*., 2016; Morris *et al.*, 2016; Petrin *et al.*, 2017; Nhim *et al.*, 2018). Thus, PCPs commonly recommend general diet and physical activity changes rather than specific obesity treatment and follow-up plans (Bardia *et al.*, 2007; Alexander *et al.*, 2011; van Dillen *et al*., 2013; Tseng *et al*., 2017). Most PCPs, for example, seldom prescribe anti-obesity medications (Saxon *et al.*, 2019) or initiate bariatric surgery referrals (Falvo *et al*., 2018; Pearce *et al*., 2019). Additionally, most general practice settings lack population health management strategies to identify and support individuals who do not meet weight loss goals (Baer *et al*., 2020). Yet, such strategies could be an important way to optimize patients’ weight loss outcomes, as individuals who do not achieve early weight loss (eg, within 12 weeks) with one approach are unlikely to lose weight without additional support or a completely different treatment (Elfhag & Rössner, 2010; Handjieva-Darlenska *et al.*, 2010; Kong *et al.*, 2010; Waring *et al.*, 2014; Miller *et al*., 2015; Unick *et al.*, 2015; Fitzpatrick *et al.*, 2018; Tronieri *et al.*, 2018).

To close knowledge gaps and more effectively help patients lose weight, a growing number of physicians – primarily those specialized in Internal Medicine and Family Medicine – are obtaining certification in obesity medicine through the American Board of Obesity Medicine (ABOM) (Kushner *et al*., 2017). The ABOM was established in 2011 to advance the delivery of evidence-based obesity care. Physicians who complete specific educational requirements and pass a standardized examination become ABOM Diplomates (hereafter referred to as ‘Diplomates’). In 2020, there were 4,152 Diplomates throughout the United States, and 65% were either Family Medicine or Internal Medicine providers (Stats and Data, n.d.). Diplomates are trained to deliver guideline-adherent obesity management care using a broad range of evidence-based resources (eg, anti-obesity pharmacotherapy, nutrition therapy, and bariatric surgery referral) (Gudzune *et al*., 2021). ABOM Diplomates may be a valuable resource in primary care settings, particularly if their expertise can be extended to support other PCPs and their many patients with obesity.

One potential strategy to extend the reach of ABOM Diplomate expertise may be through a team-based, collaborative care approach in which ABOM Diplomates serve as expert consultants to enhance PCPs’ delivery of evidence-based obesity care. Collaborative Care Models (CCMs) are widely used in primary care settings to improve outcomes among patients with other complex chronic conditions through patient self-management support, provider guideline dissemination and education, delivery system redesign such as team-based care, and use of population health management strategies using clinical informatics (Coleman *et al*., 2009; Yeoh *et al*., 2018). Building upon these components, the highly effective CCM for mental health conditions aims to improve outcomes for patients with depression by integrating mental health experts into the primary care team (Press *et al.*, 2017). Specifically, patients work with primary care team members (eg, PCPs, care managers) to develop initial treatment plans. Care managers then use population health management tools proactively to monitor patients’ symptoms (eg, change in depression scores) and facilitate timely intervention such as PCP follow-up or consultation with a mental health provider for patients who may need additional support (Press *et al*., 2017).

Little is known about the potential weight loss effectiveness of a collaborative care approach to obesity treatment (Ma *et al*., 2019; Lv *et al*., 2020). Prior work testing a collaborative care approach for patients with depression and obesity demonstrated modest weight loss at 12 months, though the majority of participants did not achieve clinically significant weight loss (Ma *et al*., 2019; Pagoto *et al*., 2013). Suboptimal weight loss may have been due, in part, to use of a one-size-fits-all lifestyle change program rather than personalized obesity treatment and follow-up plans (Lv *et al*., 2020).

Our team aims to develop and pilot test an innovative care model – the Weight Navigation Program (WNP) – to integrate Diplomates into primary care settings, enhance delivery of personalized obesity treatment, and successfully help more patients to achieve ≥5% body weight loss (versus traditional care models) while reducing burden on individual PCPs. The aim of this paper is to describe the design, rationale, and planned evaluation for the WNP. We hypothesize that the program will be feasible and acceptable among patients and providers. We also hypothesize that patients who engage in the WNP will achieve greater weight loss than a contemporaneous matched cohort of patients from another primary care clinic with similar sociodemographic characteristics.

## Methods

This study was approved by the University of Michigan Institutional Review Board. Funding to support this clinical-research initiative was provided by the University of Michigan’s Elizabeth Weiser Caswell Diabetes Institute (CDI), Michigan Center for Diabetes and Translational Research Pilot and Feasibility Grant Program (D.H.G; 5 P30DK092926-09), and the National Institutes of Health National Institute of Diabetes and Digestive and Kidney Diseases (D.H.G; 1 K23 DK123416-01A1). Additionally, the Division of Metabolism, Endocrine, and Diabetes (MEND) provided clinic space and support staff, and the Department of Family Medicine supported one faculty member’s half-day per week commitment to this pilot program.

### WNP team members and stakeholders

The Weight Navigation Program (WNP) team consists of an Endocrinologist (AK; Program Director), a Family Medicine physician and ABOM Diplomate (AO; Medical Director), an Internal Medicine physician-researcher and ABOM Diplomate (DHG; Research Director), a clinical project manager (CD), a research project manager, a clinic scheduler, and a medical assistant. The Program Director interfaces with institutional stakeholders, including representatives from the programs shown in Table 1, coordinates personnel training, and directs program implementation. The Medical Director interfaces with PCP colleagues, leads informational talks about the WNP and referral criteria, and provides clinical care for WNP patients; she is referred to as the Weight Navigator (WN) hereafter. The Research Director leads efforts to refine and evaluate the effects of the WNP.

**Table 1.** A summary of locally available weight management resources

Health system resources to support weight control	
Nutrition Services	Registered Dietitians (RD) are available to provide personalized diet recommendations
MHealthy	Available to health system-employed faculty and staff Includes a range of classes, programs, and resources that assist with lifestyle improvement efforts (eg, eating habits, weight control, and healthy lifestyle change)
Metabolic Fitness Program	Offered through Preventative Cardiology Provides a cardiovascular disease risk assessment and then provides exercise, nutrition, stress management, and behavior change strategies to risk Group-based program with virtual option for participation 24 weeks in duration
Obesity & Metabolic Disorders Program	Offered through The Division of Metabolism, Endocrinology, and Diabetes to patients with obesity and at least one other metabolic disorder (eg, type 2 diabetes, polycystic ovarian syndrome) Includes a comprehensive history, physical, genetic screening, and laboratory assessment to guide individualized and multi-component treatment plans.
Weight Management Program	Evidence-based, medically supervised, intensive medical behavioral program that utilizes meal replacement 2 or more years in duration Individuals work with endocrinologists and dietitians to achieve and maintain ≥10%–15% weight loss
Endoscopic Bariatric Therapy	Endoscopic procedures performed by gastroenterologists with specialized training Designed for individuals who are not candidates for weight loss surgery or who prefer a less-invasive, non-surgical alternative.
Bariatric surgery	Laparoscopic sleeve gastrectomy or Roux-en-Y-gastric bypass options are available. Michigan Medicine program consists of a multidisciplinary care team that includes surgeons, physician assistants, registered dietitians, neuropsychologists, endocrinologists, and nurses; all team members have completed specialized training.
Post-bariatric Endocrinology	All patients undergoing bariatric surgery at the institution are offered ‘lifelong’ follow-up in the long-term care clinic. The clinic also serves as a referral center for patients experiencing long-term complications even if the surgery was performed elsewhere. Team of endocrinologists and dietitians with expertise in obesity and bariatric care who conduct medical assessments, laboratory monitoring (eg, vitamin/mineral levels), behavior change counseling, and initiation of weight loss pharmacotherapy medical or subspecialty referrals, if needed
Resource to support diagnosis of weight-related conditions and inform treatment plan recommendations	
Non-Alcoholic Fatty Liver Disease (NALFD) Clinic	Hepatologists diagnose NAFLD, monitor patients over time, and provide treatment recommendations, which include weight loss and dietary modification.
Lipodystrophy and Atypical Diabetes Clinic:	Special expertise provided by the endocrinologist in the Lipodystrophy/Atypical Diabetes Clinic allows for the identification and treatment of these rare conditions.
Obesity hypoventilation clinic	Sleep Medicine specialists can diagnosis this condition and recommend specific treatment
Metabolism, Endocrinology & Diabetes (MEND) Clinic	Endocrinologists in the MEND clinic screen for secondary hormonal causes of obesity.

Key representatives from the weight management and evaluation subspecialty groups shown in Table 1 facilitated a brief training experience for the Weight Navigator to gain knowledge in the full spectrum of health system and community weight management resources. Over the course of 3 months, the Weight Navigator spent time with team members from the various groups to learn the details of each program’s eligibility criteria, goals, and operations, thus enabling her to better guide patients’ informed treatment choices.

### Study design

This is a clinical quality improvement project with an embedded single-arm pilot study. Specifically, patients with obesity who desire to lose weight will be referred by their PCP to the WNP. Patients scheduled for a WNP appointment will be offered the opportunity to participate in research; we will aim to recruit 30 participants to the research arm of the study Research activities include (1) surveys at 0, 6, and 12 months; (2) optional semi-structured interview participation at 0 and 6 months; and (3) use of a text messaging program for remote weight monitoring.

### Study setting

Michigan Medicine is a large, academic medical center that includes 14 adult primary care clinics throughout southeast Michigan that serve approximately 240 000 patients. The pilot Weight Navigation Program will be initiated at a single Michigan Medicine primary care site. Michigan Medicine also offers diverse resources for the evaluation and management of obesity and obesity-related chronic conditions (Table 1). Despite the availability of these programs, unpublished survey data of PCPs in our health system (*n* = 107) demonstrate that most providers refer patients with obesity to a dietitian (*n* = 89, 83%) while only a minority utilize other resources such as medical weight management (*n* = 23, 21.5%) or weight loss surgery (*n* = 20, 18.7%).

### Participants WNP eligibility

Primary care patients with a body mass index (BMI) ≥ 30 kg/m^2^ and at least one weight-related chronic condition (eg, type 2 diabetes, hypertension, hyperlipidemia, non-alcoholic fatty liver disease, obstructive sleep apnea) may be referred by their PCP to the WNP. WNP exclusion criteria include primary care received at a location other than the pilot clinic site and pregnancy or breastfeeding. Patients may be scheduled for a WNP appointment regardless of insurance type. However, patients’ eligibility and out-of-pocket costs for obesity treatments may differ based on insurance status; this will be taken into consideration by the Weight Navigator when working with patients to develop personalized treatment plans.

### Pilot research program eligibility

Eligibility criteria included the following: (1) scheduled for a WNP; (2) willingness to complete surveys at 0, 6, and 12 months; (3) willingness and ability to self-report weight data by text message; and (4) willingness to receive outreach from the study team in response to text messaging data. Exclusion criteria are the inability to read or write English.

### Pilot research program eligibility

Eligibility criteria included the following: (1) scheduled for a WNP; (2) willingness to complete surveys at 0, 6, and 12 months; (3) willingness and ability to self-report weight data by text message; and (4) willingness to receive outreach from the study team in response to text messaging data. Exclusion criteria are the inability to read or write English.

### Recruitment

Patients will be referred to the WNP by PCPs. All patients scheduled for a WNP appointment will be eligible for participation in a single-arm, 12-month pilot study. We anticipate that the Weight Navigator will see approximately 4 patients per 4-h clinic session once per week. Accounting for vacations, holidays, and canceled appointments, we estimate that the Weight Navigator will see approximately 150 patients during the first year of the program.

All patients scheduled for a WNP appointment will be eligible for participation in a single-arm, 12-month pilot research study. A research project manager will contact scheduled patients by telephone at least 5 days prior to their appointment date to invite them to participate in research and describe the study processes. Individuals who desire to participate in research will complete an online informed consent document and the baseline survey prior to the WNP appointment using the RedCap survey platform (REDCap, n.d.). Recruitment will continue until we reach our target of 30 participants.

### Intervention

**Figure 1.**
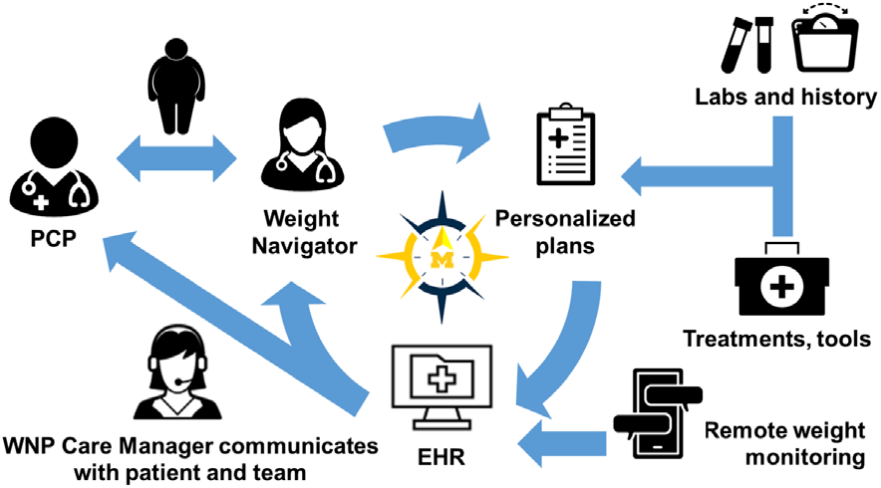
Weight Navigation Program (WNP) design. Patients with obesity and ≥1 weight-related condition who desire to lose weight are referred by their primary care providers (PCPs) to the WNP. The Weight Navigator and patient develop a personalized obesity treatment plan using existing health system, community, and pharmacotherapeutic resources. The plan is communicated to PCPs via the Electronic Health Record (EHR). Patients self-report weight data using an EHR-based text messaging platform. The WNP team is notified of patients’ weight changes according to pre-specific thresholds. The WNP Care Manager initiates tailored outreach to support patients over time, address potential barriers, and facilitate changes to the treatment plan, if needed, to optimize patients’ outcomes.

The WNP model draws on principles of the collaborative care model (CCM), (Woltmann *et al*., 2012; Miller *et al*., 2013; Press *et al*., 2017) personalized medicine (Yanovski & Yanovski, 2018), and population health management(Neuwirth *et al.*, 2007) to enhance the delivery of team-based, patient-centered, outcome-driven obesity care. Figure 1 shows a conceptual overview of the WNP, which is described in detail below.

Eligible patients who desire to lose weight may be referred to the WNP by their PCP. Referral orders will be reviewed by a clinical scheduler who will confirm patient eligibility, contact patients to schedule an appointment, and send patients a pre-visit weight history questionnaire via the patient Electronic Health Record (EHR) portal in advance of their appointment; weight history questionnaire topics are shown in Table 2. To ensure the Weight Navigator has access to relevant clinical data at the time of the WNP encounter, the referral order will prompt PCPs to obtain a comprehensive metabolic panel, complete blood count, hemoglobin HA1c, fasting glucose, fasting lipid panel, and thyroid function tests.

**Table 2.** Weight history questionnaire topics

Birth history and birth weight Weight changes over time (eg, highest and lowest weights) Food frequency assessment (eg, frequency of eating fast food, processed food, high-fat food, sweets) Eating patterns and timing (eg, number of meals and snacks per day binge eating, night eating) Eating triggers Monthly food budget Prior weight loss successes and challenges Physical activity habits

During a 1-h appointment, the Weight Navigator will review patients’ medical history, weight history, co-morbidities, and laboratory data. The Weight Navigator may identify potential obesogenic conditions (eg, hypothyroidism), obesogenic medications (eg, insulin), obesity-related conditions (eg, non-alcoholic fatty liver disease), and other co-morbidities that may hinder weight loss (eg, untreated depression). Such observations and management recommendations will be communicated to patients during the visit and to their PCPs via the EHR. Clinical assessment and review of laboratory data will also allow the Weight Navigator to determine whether additional evaluation is necessary (eg, secondary hormonal workup).

The Weight Navigator and patient will review available health system, community, and pharmacologic obesity treatments and together develop a plan that is responsive to the patient’s individual experiences, current preferences, and financial constraints. The Weight Navigation will place initial treatment referrals. In the visit encounter note, the Weight Navigator will also detail other treatment modalities that the PCP may consider in the future, if needed. Moreover, if a patient desires to try an anti-obesity medication, the Weight Navigator will make a treatment recommendation and detail the medication titration schedule in the visit note. The Weight Navigator will also provide information to patients and their PCPs regarding the potential out-of-pocket medication costs and availability of drug discount programs.

Visits will be billed using the 99 215 Evaluation and Management (E/M) code for a 60-min visit. The 99 417 E/M code may be added if the provider spends an additional 15 min on same-day documentation.

To optimize patients’ achievement of ≥5% body weight loss, we will test a population health management strategy to support individuals over time and specifically identify and support early weight loss non-responders (eg, those that do not achieve ≥3% body weight loss within 12 months). Research participants will be invited to self-report weight data via the Michigan Patient Outreach Texting Application (MPOTA), a text messaging platform integrated with Michigan Medicine’s EHR (EPIC) that allows for the remote collection of patient-reported data. We worked with Michigan Medicine’s Virtual Care Department to adapt MPOTA for Weight Management (MPOTA-WM).

MPOTA-WM participants will be asked to self-weigh at least once per week and report their weight data via text message. Participants without access to a home scale will be provided with one by the study team. Participants will be asked to use the same scale throughout the duration of the study. Participants will self-select the day(s) of the week and time of the day to receive the following text message: ‘Hello, this is your Weight Navigation Program Research Team. What was your weight in pounds today? Reply STOP to opt out of receiving messages from our team’. The WNP team will receive inbox notifications based on the following parameters, which have been adapted from prior literature (Baer *et al*., 2020): (1) weight gain of ≥ 3 pounds at any point; (2) weight loss of varying thresholds (eg, 3%, 5%, and 10% body weight loss); (3) non-response to MPOTA for weight management messages for 14 days; and (4) participants’ ‘STOP’ responses, indicating that they are discontinuing their participation in the text messaging program. The research project manager will initiate tailored outreach; those losing weight, for example, will receive a supportive EHR portal message while those gaining weight will receive a phone call to discuss barriers to weight management, individuals’ preferences and needs, and other available weight management options, as detailed in the WNP encounter note. Communication will be documented in the EHR and patients’ primary care, WNP, and/or weight management subspecialty providers will be alerted, as appropriate.

### Outcome measures

We have 4 outcome categories: (1) feasibility and acceptability of the WNP; (2) clinical impact of the WNP; (3) feasibility, acceptability, and preliminary effectiveness of adding remote weight monitoring by text messaging to the WNP; and (4) feasibility and acceptability of research processes including recruitment and data collection.

#### Feasibility and acceptability of the WNP

Consistent with recommendations for conducting pilot studies (Eldridge *et al.*, 2016), our primary outcomes will be measures of feasibility (ie, extent to which an innovation can be successfully used in a given setting) and acceptability (ie, stakeholder perception that an innovation is satisfactory). Measures of WNP feasibility will include rates of (1) referral (ie, number of patients referred to the WNP divided by the number of patients eligible for the program, as determined by data extraction from the EHR) and (2) uptake (ie, number of patients referred to the program divided by the number of individuals who complete a WNP appointment).

We will assess WNP intervention acceptability through semi-structured interviews with a purposive sample of WNP research program participants to explore individuals’ experiences with the program and solicit feedback on opportunities for improvement. We will also survey primary care providers and patients regarding their satisfaction with the program and solicit feedback on opportunities for improvement.

#### Clinical impact of the WNP

##### Change in weight

We will abstract weight and height data from the EHR for all WNP patients who complete a clinic visit within the first 12 months of clinical program operations. We will evaluate average weight change among WNP patients during the 12 months following their WNP appointment. We will also identify a contemporaneous cohort of patients with obesity matched by gender and approximate age (within 10 years) to WNP patients. The matched cohort will be selected from another Michigan Medicine primary care clinic located less than 1 mile from the pilot site clinic and serving a similar patient population. We will compare between-group weight change among WNP patients and the matched cohort. In addition to evaluating mean weight change, we will also evaluate achievement of ≥5% body weight loss.

##### Referrals to health system weight management resources

We will compare the number of WNP referrals to health system weight management resources to the number of referrals by PCPs of patients in the matched cohort.

##### Patients’ engagement with health system weight management resources

We will compare the rate of engagement in health system weight management resources among WNP patients as compared to patients in the matched cohort.

##### Prescriptions for anti-obesity medications

We will compare the number of WNP prescriptions for anti-obesity medications to the number of prescriptions by PCPs of patients in the matched cohort.

##### Patients’ use of anti-obesity medications

We will compare the number of refills for anti-obesity medications among WNP patients to patients in the matched cohort.

##### Change in survey measures

Research program participants will be asked to complete surveys at baseline, 6 months, and 12 months; we will determine changes in measures at 6 and 12 months compared to baseline. Survey measures include motivation to lose weight (Kullgren, 2016), perceived competence to lose weight (Williams *et al*., 1998), social support (Sarason *et al.*, 1987), and eating behaviors (de Lauzon *et al.*, 2004). At baseline, we will ask participants to report sociodemographic characteristics.

### Feasibility, acceptability, and preliminary effectiveness of adding remote weight monitoring by text messaging to the WNP

#### MPOTA-WM uptake

Determined by the number of individuals who consent to research divided by the number of individuals who self-report at least 1 weight measure by text message.

#### MPOTA-WM engagement

Determined by the number of self-reported weights divided by the total number of possible reporting days.

#### Qualitative experience with MPOTA-WM

During interviews with research program participants, we will also explore individuals’ experiences with MPOTA-WM and solicit feedback on opportunities to improve the text messaging program.

#### Change in weight

We will evaluate change in weight from baseline to 12 months of follow-up among MPOTA-WM users. To account for potential differences between clinic and home scales, the first weight entered by text message will serve as participants’ baseline weight for remote weight monitoring and this will be compared with follow-up MPOTA-WM data.

### Feasibility and acceptability of research processes including recruitment and data collection

#### Rate of research recruitment

Determined by the number of individuals who consent to research divided by the total number of individuals invited to participate in research divided by the number of days in the recruitment period.

#### Research retention

Determined by the number of research participants who complete surveys at 6 and 12 months divided by the total number of research participants.

### Data analyses

#### Quantitative data analysis

Measures of central tendency (eg, proportions, means, standard deviations) will be used for all descriptive analyses. We will calculate mean 3-month and 6-month weight changes from baseline. We will also calculate the number of participants that achieve ≥5% body weight loss at 6 and 12 months. We will compare between-group changes (ie, all WNP participants versus WNP research participants and all WNP participants versus matched cohort) in weight using a difference-in-difference analytic approach. Among WNP research participants, we will also calculate mean weight change based on MPOTA-WM data. We will conduct all analyses using Stata 15.

#### Qualitative data analysis

Semi-structured interviews will be recorded and transcribed verbatim. Interviews will be imported into qualitative analysis software, Dedoose (*n.d.), and analyzed using template qualitative analysis (Brooks *et al*., 2015). The initial codebook will be developed to reflect interview questions, and additional codes will be subsequently generated to reflect new concepts that emerge from the data. Two trained coders will independently review and code each transcript and then meet to resolve coding differences and iteratively revise the codebook.*

#### Integrated analysis

Consistent with a mixed-methods sequential explanatory design (Ivankova, 2006), we will integrate (ie, connect) the quantitative and qualitative findings in the final stage of data analysis. In this way, we will interpret our quantitative data in the context of qualitative participant experience.

## Discussion

We describe the design, rationale, and study protocol for a quality improvement initiative with an embedded single-arm pilot study. The WNP aims to overcome the known challenges of providing evidence-based obesity management in general practice settings. This model consists of three components that have demonstrated effectiveness when used independently for other primary care improvement efforts: (1) team-based collaborative care; (2) personalized weight management plans; and (3) population health management to optimize patients’ outcomes.

### Limitations and opportunities

This program has several potential limitations. First, it will be implemented at a single site and thus may not be generalizable to all practice settings. However, we believe the WNP model offers a conceptual and practical framework that may be successfully adapted to diverse primary settings where at least one physician is certified in obesity medicine. Second, cost barriers may impede patients’ use of certain weight management resources. Fortunately, there is growing insurance coverage for weight management services (Jannah *et al.*, 2018), drug discount programs support the use of some anti-obesity medications (Prescription Prices, Coupons & Pharmacy Information – GoodRx, n.d.), and most health plans cover nutrition counseling services. Moreover, we will utilize low-cost, community-based resources such as Diabetes Prevention Programs when developing personalized obesity treatment plans. Fourth, some components of the current WNP model (eg, MPOTA-WM outreach) require research funding, which may limit implementation in general practice settings. However, to the extent possible, the WNP model uses billable services, and we will aim to identify opportunities to integrate MPOTA-WM into routine clinical care. Moreover, remote monitoring tools are now common features of many EHR systems, reimbursement guidelines encourage use of such tools (Final Policy, Payment, and Quality Provisions Changes to the Medicare Physician Fee Schedule for Calendar Year 2021 | CMS, (n.d.), and additional policy changes on the horizon may enable more providers to play active roles supporting patients with obesity (Cassidy, 2019).

### Future directions

We plan to evaluate the pilot WNP using mixed quantitative and qualitative data collection methods. These data will enable us to evaluate the program’s feasibility, acceptability, and preliminary weight loss effectiveness. Moreover, we will use qualitative data obtained during interviews with patients to identify the features of the program that work well and those that could be improved. These data will enable us to refine the program and test its effectiveness in diverse practice settings. We anticipate evaluating the refined program in a future large-scale pragmatic trial using an effectiveness-implementation hybrid type 1 design to evaluate the program’s weight loss effectiveness at 12 and 24 months while also gathering information on implementation processes and determinants (Curran *et al.*, 2012).

## Conclusions

Obesity is a complex chronic condition that demands evidence-based, personalized, and longitudinal care. To deliver such care in general practice, the WNP leverages ABOM Diplomate expertise, health system and community weight management resources, and EHR-based population health management tools. Moreover, to the extent possible, the WNP uses existing weight management resources and billable services to help patients develop personalized weight management plans. Thus, the model may provide an effective, sustainable, and scalable opportunity to improve obesity management in primary care settings.

## References

[ref2] Alexander SC , Cox ME , Boling Turer CL , Lyna P , Østbye T , Tulsky JA , Dolor RJ and Pollak KI (2011) Do the five A’s work when physicians counsel about weight loss? Family Medicine 43, 179–184. 21380950 PMC3367376

[ref3] Anderson JW and Konz EC (2001) Obesity and disease management: effects of weight loss on comorbid conditions. Obesity Research 9 (Suppl 4), 326S–334S. 11707561 10.1038/oby.2001.138

[ref4] Baer HJ , De La Cruz BA , Rozenblum R , Nolido NV , Orav EJ , Metzler K , Block JP , Halperin F , McManus KD , Aronne, LJ , Minero G and Bates DW (2020) Integrating an online weight management program with population health management in primary care: design, methods, and baseline data from the PROPS randomized controlled trial (partnerships for reducing overweight and obesity with patient-centered strategies). Contemporary Clinical Trials 95, 106026. 32428586 10.1016/j.cct.2020.106026

[ref5] Bardia A , Holtan SG , Slezak JM and Thompson WG (2007) Diagnosis of obesity by primary care physicians and impact on obesity management. Mayo Clinic Proceedings 82, 927–932. 17673060 10.4065/82.8.927

[ref6] Bernstein KM , Manning DA and Julian RM (2016) Multidisciplinary teams and obesity: role of the modern patient-centered medical home. Primary Care 43, 53–59. 26896199 10.1016/j.pop.2015.08.010

[ref7] Brooks J , McCluskey S , Turley E and King N (2015) The utility of template analysis in qualitative psychology research. Qualitative Research in Psychology 12, 202–222. 27499705 10.1080/14780887.2014.955224 PMC4960514

[ref8] Cabana MD , Rand CS , Powe NR , Wu AW , Wilson MH , Abboud PA and Rubin HR (1999) Why don’t physicians follow clinical practice guidelines? A framework for improvement. JAMA 282, 1458–1465. 10535437 10.1001/jama.282.15.1458

[ref9] Cassidy B (2019) S.595 – 116th Congress (2019–2020): treat and reduce obesity act of 2019. Retrieved 12 January 2022 from https://www.congress.gov/bill/116th-congress/senate-bill/595

[ref10] Cawley J , Meyerhoefer C , Biener A , Hammer M and Wintfeld N (2015) Savings in medical expenditures associated with reductions in body mass index among US adults with obesity, by diabetes status. Pharmacoeconomics 33, 707–722. 25381647 10.1007/s40273-014-0230-2 PMC4486410

[ref11] Coleman K , Austin BT , Brach C and Wagner EH (2009) Evidence on the chronic care model in the new millennium. Health Affairs (Project Hope) 28, 75–85. 19124857 10.1377/hlthaff.28.1.75 PMC5091929

[ref12] Curran GM , Bauer M , Mittman B , Pyne JM and Stetler C (2012) Effectiveness-implementation hybrid designs. Medical Care 50, 217–226. 22310560 10.1097/MLR.0b013e3182408812 PMC3731143

[ref13] de Lauzon B , Romon M , Deschamps V , Lafay L , Borys J-M , Karlsson J , Ducimetière P , Charles MA and Fleurbaix Laventie Ville Sante Study Group (2004) The three-factor eating questionnaire-R18 is able to distinguish among different eating patterns in a general population. The Journal of Nutrition 134, 2372–2380. 15333731 10.1093/jn/134.9.2372

[ref14] Diabetes Prevention Program Research Group (2009) 10-Year follow-up of diabetes incidence and weight loss in the diabetes prevention program outcomes study. The Lancet 374, 1677–1686. 10.1016/S0140-6736(09)61457-4 PMC3135022 19878986

[ref15] Eldridge SM , Chan CL , Campbell MJ , Bond CM , Hopewell S , Thabane L and Lancaster GA (2016) CONSORT 2010 statement: extension to randomised pilot and feasibility trials. Pilot and Feasibility Studies 2, 64. 27965879 10.1186/s40814-016-0105-8 PMC5154046

[ref16] Elfhag K and Rössner S (2010) Initial weight loss is the best predictor for success in obesity treatment and sociodemographic liabilities increase risk for drop-out. Patient Education and Counseling 79, 361–366. 20223613 10.1016/j.pec.2010.02.006

[ref17] Falvo AM , Hite Philp F and Eid GM (2018) Primary care provider management of patients with obesity at an integrated health network: a survey of practices, views, and knowledge. Surgery for Obesity and Related Diseases: Official Journal of the American Society for Bariatric Surgery 14, 1149–1154. 29929858 10.1016/j.soard.2018.05.002

[ref18] Final Policy, Payment, and Quality Provisions Changes to the Medicare Physician Fee Schedule for Calendar Year 2021 | CMS (n.d.) Newsroom. Retrieved 19 January 2021 from https://www.cms.gov/newsroom/fact-sheets/final-policy-payment-and-quality-provisions-changes-medicare-physician-fee-schedule-calendar-year-1

[ref19] Final Recommendation Statement: Weight Loss to Prevent Obesity-Related Morbidity and Mortality in Adults: Behavioral Interventions - US Preventive Services Task Force (2003) Retrieved 26 January 2017 from https://www.uspreventiveservicestaskforce.org/uspstf/document/RecommendationStatementFinal/obesity-in-adults-interventions 10.1001/jama.2018.13022 30326502

[ref20] Fitzpatrick SL , Appel LJ , Bray B , Brooks N and Stevens VJ (2018) Predictors of long-term adherence to multiple health behavior recommendations for weight management. Health Education & Behavior: The Official Publication of the Society for Public Health Education 45, 997–1007. 29478353 10.1177/1090198118757823 PMC6133769

[ref21] Foster GD , Wadden TA , Makris AP , Davidson D , Sanderson RS , Allison DB and Kessler A (2003) Primary care physicians’ attitudes about obesity and its treatment. Obesity Research 11, 1168–1177. 14569041 10.1038/oby.2003.161

[ref22] Francis JJ , Johnston M , Robertson C , Glidewell L , Entwistle V , Eccles MP and Grimshaw JM (2010) What is an adequate sample size? Operationalising data saturation for theory-based interview studies. Psychology & Health 25, 1229–1245. 20204937 10.1080/08870440903194015

[ref23] Garvey WT , Ryan DH , Look M , Gadde KM , Allison DB , Peterson CA , Schwiers M , Day WW and Bowden CH (2012) Two-year sustained weight loss and metabolic benefits with controlled-release phentermine/topiramate in obese and overweight adults (SEQUEL): a randomized, placebo-controlled, phase 3 extension study. The American Journal of Clinical Nutrition 95, 297–308. 22158731 10.3945/ajcn.111.024927 PMC3260065

[ref24] Gudzune KA , Wickham EP , Schmidt SL and Stanford FC (2021) Physicians certified by the American board of obesity medicine provide evidence-based care. Clinical Obesity 11, e12407. 33280270 10.1111/cob.12407 PMC9999726

[ref25] Handjieva-Darlenska T , Handjiev S , Larsen TM , van Baak MA , Jebb S , Papadaki A , Pfeiffer AFH , Martinez JA , Kunesova M , Holst C , Saris WHM and Astrup A (2010) Initial weight loss on an 800-kcal diet as a predictor of weight loss success after 8 weeks: the Diogenes study. European Journal of Clinical Nutrition 64, 994–999. 20588292 10.1038/ejcn.2010.110

[ref26] Home | Dedoose (n.d.) Retrieved 11 December 2018 from https://www.dedoose.com/

[ref27] Ivankova NV (2006) Using mixed-methods sequential explanatory design: from theory to practice. Field Methods 18, 3–20.

[ref28] Jannah N , Hild J , Gallagher C and Dietz W (2018) Coverage for obesity prevention and treatment services: analysis of Medicaid and state employee health insurance programs. Obesity (Silver Spring, Md.) 26, 1834–1840. 10.1002/oby.22307 30426721

[ref29] Jensen MD , Ryan DH , Apovian CM , Ard JD , Comuzzie AG , Donato KA , Hu FB , Hubbard VS , Jakicic JM , Kushner RF , Loria CM , Millen BE , Nonas CA , Pi-Sunyer FX , Stevens J , Stevens VJ , Wadden TA , Wolfe BM , Yanovski SZ , Jordan HS , Kendall KA , Lux LJ , Mentor-Marcel R , Morgan LC , Trisolini MG , Wnek J , Anderson JL , Halperin JL , Albert NM , Bozkurt B , Brindis RG , Curtis LH , DeMets D , Hochman JS , Kovacs RJ , Ohman EM , Pressler SJ , Sellke FW , Shen WK , Smith SC Jr , Tomaselli GF (2014) 2013 AHA/ACC/TOS guideline for the management of overweight and obesity in adults. Circulation 12 (Suppl 2), S102–S138. 10.1161/01.cir.0000437739.71477.ee PMC5819889 24222017

[ref30] Kahan S and Zvenyach T (2016) Obesity as a disease: current policies and implications for the future. Current Obesity Reports 5, 291–297. 27099165 10.1007/s13679-016-0218-7

[ref31] Kong W , Langlois M-F , Kamga-Ngandé C , Gagnon C , Brown C and Baillargeon J-P (2010) Predictors of success to weight-loss intervention program in individuals at high risk for type 2 diabetes. Diabetes Research and Clinical Practice 90, 147–153. 20655608 10.1016/j.diabres.2010.06.031

[ref32] Kullgren JT (2016) *A mixed methods study of initial engagement in behaviors to prevent diabetes among employees with prediabetes*. Society of General Internal Medicine Annual Meeting 2016 (Oral Presentation). Hollywood, FL.

[ref33] Kushner RF , Brittan D , Cleek J , Hes D , English W , Kahan S and Aronne LJ (2017) The American board of obesity medicine: 5-year report. Obesity 25, 982–984. 28544791 10.1002/oby.21828

[ref34] Lv N , Xiao L , Majd M , Lavori PW , Smyth JM , Rosas LG , Venditti EM , Snowden MB , Lewis MA , Ward E , Lesser L , Williams LM , Azar KMJ and Ma J (2020) Variability in engagement and progress in efficacious integrated collaborative care for primary care patients with obesity and depression: within-treatment analysis in the RAINBOW trial. PLoS One 15, e0231743. 32315362 10.1371/journal.pone.0231743 PMC7173791

[ref35] Ma J , Rosas LG , Lv N , Xiao L , Snowden MB , Venditti EM , Lewis MA , Goldhaber-Fiebert JD and Lavori PW (2019) Effect of integrated behavioral weight loss treatment and problem-solving therapy on body mass index and depressive symptoms among patients with obesity and depression: the RAINBOW randomized clinical trial. JAMA 321, 869–879. 30835308 10.1001/jama.2019.0557 PMC6439596

[ref36] Mainous AG , Tanner RJ and Baker R (2016) Prediabetes diagnosis and treatment in primary care. Journal of the American Board of Family Medicine: JABFM 29, 283–285. 26957387 10.3122/jabfm.2016.02.150252

[ref37] Miller CJ , Grogan-Kaylor A , Perron BE , Kilbourne AM , Woltmann E and Bauer MS (2013) Collaborative chronic care models for mental health conditions: cumulative meta-analysis and metaregression to guide future research and implementation. Medical Care 51, 922–930. 23938600 10.1097/MLR.0b013e3182a3e4c4 PMC3800198

[ref38] Miller CK , Nagaraja HN and Weinhold KR (2015) Early weight-loss success identifies nonresponders after a lifestyle intervention in a worksite diabetes prevention trial. Journal of the Academy of Nutrition and Dietetics 115, 1464–1471. 26095435 10.1016/j.jand.2015.04.022 PMC4554978

[ref39] Morris GL , Chapman K , Nelson D , Fink J , Walker R and Cisler RA (2016) Physician use of electronic health records in obesity management. WMJ: Official Publication of the State Medical Society of Wisconsin 115, 140–142. 27443090

[ref40] Neuwirth EEB , Schmittdiel JA , Tallman K and Bellows J (2007) Understanding panel management: a comparative study of an emerging approach to population care. The Permanente Journal 11, 12–20. 10.7812/tpp/07-040 PMC3057714 21461107

[ref41] Nhim K , Khan T , Gruss SM , Wozniak G , Kirley K , Schumacher P , Luman ET and Albright A (2018) Primary care providers’ prediabetes screening, testing, and referral behaviors. American Journal of Preventive Medicine 55, e39–e47. 29934016 10.1016/j.amepre.2018.04.017 PMC6241213

[ref42] Ossolinski G , Jiwa M and McManus A (2015) Weight management practices and evidence for weight loss through primary care: a brief review. Current Medical Research and Opinion 31, 2011–2020. 26288258 10.1185/03007995.2015.1082993

[ref43] Pagoto S , Schneider KL , Whited MC , Oleski JL , Merriam P , Appelhans B , Ma Y , Olendzki B , Waring ME , Busch AM , Lemon S , Ockene I and Crawford S (2013) Randomized controlled trial of behavioral treatment for comorbid obesity and depression in women: the be active trial. International Journal of Obesity (2005) 37, 1427–1434. 23459323 10.1038/ijo.2013.25 PMC3675166

[ref44] Pearce C , Rychetnik L , Wutzke S and Wilson A (2019) Obesity prevention and the role of hospital and community-based health services: a scoping review. BMC Health Services Research 19, 453. 31277640 10.1186/s12913-019-4262-3 PMC6612151

[ref45] Petrin C , Kahan S , Turner M , Gallagher C and Dietz WH (2017) Current attitudes and practices of obesity counselling by health care providers. Obesity Research & Clinical Practice 11, 352–359. 27569863 10.1016/j.orcp.2016.08.005

[ref46] Phelan SM , Burgess DJ , Yeazel MW , Hellerstedt WL , Griffin JM and van Ryn M (2015) Impact of weight bias and stigma on quality of care and outcomes for patients with obesity. Obesity Reviews: An Official Journal of the International Association for the Study of Obesity 16, 319–326. 25752756 10.1111/obr.12266 PMC4381543

[ref47] Popkin, BM , Du S , Green WD , Beck MA , Algaith T , Herbst CH , Alsukait RF , Alluhidan M , Alazemi N and Shekar M (2020) Individuals with obesity and COVID-19: a global perspective on the epidemiology and biological relationships. Obesity Reviews 21, e13128. 32845580 10.1111/obr.13128 PMC7461480

[ref48] Prescription Prices, Coupons & Pharmacy Information – GoodRx (n.d.) Retrieved 10 October 2019 from https://www.goodrx.com/

[ref49] Press MJ , Howe R , Schoenbaum M , Cavanaugh S , Marshall A , Baldwin L and Conway PH (2017) Medicare payment for behavioral health integration. The New England Journal of Medicine 376, 405–407. 27973984 10.1056/NEJMp1614134

[ref50] Prospective Studies Collaboration (2009) Body-mass index and cause-specific mortality in 900 000 adults: collaborative analyses of 57 prospective studies. The Lancet 373, 1083–1096. 10.1016/S0140-6736(09)60318-4 PMC2662372 19299006

[ref51] REDCap. (n.d.) Retrieved 19 June 2017 from https://www.project-redcap.org/

[ref52] Rothberg AE , McEwen LN , Kraftson AT , Ajluni N , Fowler CE , Nay CK , Miller NM , Burant CF and Herman WH (2017) Impact of weight loss on waist circumference and the components of the metabolic syndrome. BMJ Open Diabetes Research & Care 5, e000341. 10.1136/bmjdrc-2016-000341 PMC5337678 28316795

[ref53] Salinas GD , Glauser TA , Williamson JC , Rao G and Abdolrasulnia M (2011) Primary care physician attitudes and practice patterns in the management of obese adults: results from a national survey. Postgraduate Medicine 123, 214–219. 21904104 10.3810/pgm.2011.09.2477

[ref54] Saxon DR , Iwamoto SJ , Mettenbrink CJ , McCormick E , Arterburn D , Daley MF , Oshiro CE , Koebnick C , Horberg M , Young DR and Bessesen DH (2019) Antiobesity medication use in 2.2 million adults across eight large health care organizations: 2009–2015. Obesity (Silver Spring, Md.) 27, 1975–1981. 10.1002/oby.22581 PMC6868321 31603630

[ref55] Sarason IG , Sarason BR , Shearin EN and Pierce GR (1987) A brief measure of social support: practical and theoretical implications. Journal of Social and Personal Relationships 4, 497–510. 10.1177/0265407587044007

[ref56] Stats and Data (n.d.) *American Board of Obesity Medicine*. Retrieved 11 December 2020 from https://www.abom.org/stats-data-2/

[ref57] Thomas J and Harden A (2008) Methods for the thematic synthesis of qualitative research in systematic reviews. BMC Medical Research Methodology 8, 45. 18616818 10.1186/1471-2288-8-45 PMC2478656

[ref58] Tronieri JS , Wadden TA , Chao AM , Pearl RL , Alamuddin N and Berkowitz RI (2018) Early weight loss in behavioral treatment predicts later rate of weight loss and response to pharmacotherapy. Annals of Behavioral Medicine: A Publication of the Society of Behavioral Medicine. 10.1093/abm/kay036 PMC6374712 29800080

[ref59] Tsai AG and Wadden TA (2009) Treatment of obesity in primary care practice in the United States: a systematic review. Journal of General Internal Medicine 24, 1073–1079. 19562419 10.1007/s11606-009-1042-5 PMC2726879

[ref60] Tseng E , Greer RC , O’Rourke P , Yeh H-C , McGuire MM , Clark JM and Maruthur NM (2017) Survey of primary care providers’ knowledge of screening for, diagnosing and managing prediabetes. Journal of General Internal Medicine 32, 1172–1178. 28730532 10.1007/s11606-017-4103-1 PMC5653548

[ref61] Unick JL , Leahey T , Kent K and Wing RR (2015) Examination of whether early weight loss predicts 1-year weight loss among those enrolled in an Internet-based weight loss program. International Journal of Obesity (2005) 39, 1558–1560. 25982792 10.1038/ijo.2015.89 PMC4596751

[ref62] van Dillen SME , van Binsbergen JJ , Koelen MA and Hiddink GJ (2013) Nutrition and physical activity guidance practices in general practice: a critical review. Patient Education and Counseling 90, 155–169. 23246149 10.1016/j.pec.2012.10.022

[ref63] Wadden TA , Butryn ML , Hong PS and Tsai AG (2014) Behavioral treatment of obesity in patients encountered in primary care settings: a systematic review. JAMA 312, 1779. 25369490 10.1001/jama.2014.14173 PMC4443898

[ref64] Waring ME , Schneider KL , Appelhans BM , Busch AM , Whited MC , Rodrigues S , Lemon SC and Pagoto SL (2014) Early-treatment weight loss predicts 6-month weight loss in women with obesity and depression: implications for stepped care. Journal of Psychosomatic Research 76, 394–399. 24745781 10.1016/j.jpsychores.2014.03.004 PMC4038379

[ref68] Williams GC , Freedman ZR and Deci EL (1998) Supporting autonomy to motivate patients with diabetes for glucose control. Diabetes Care 21, 1644–1651. 10.2337/diacare.21.10.1644 9773724

[ref65] Woltmann E , Grogan-Kaylor A , Perron B , Georges H , Kilbourne AM and Bauer MS (2012) Comparative effectiveness of collaborative chronic care models for mental health conditions across primary, specialty, and behavioral health care settings: systematic review and meta-analysis. The American Journal of Psychiatry 169, 790–804. 22772364 10.1176/appi.ajp.2012.11111616

[ref66] Yanovski SZ and Yanovski JA (2018) Viewpoint: toward precision approaches for the prevention and treatment of obesity. JAMA 319, 223–224. 29340687 10.1001/jama.2017.20051 PMC5787370

[ref67] Yeoh EK , Wong MCS , Wong ELY , Yam C , Poon CM , Chung RY , Chong M , Fang Y , Wang HHX , Liang M , Cheung WWL , Chan CH , Zee B and Coats AJS (2018) Benefits and limitations of implementing chronic care model (CCM) in primary care programs: a systematic review. International Journal of Cardiology 258, 279–288. 29544944 10.1016/j.ijcard.2017.11.057

